# Supplementary material for: MIASurviveMTP: Machine learning for immediate assessment and survival prediction after massive transfusion protocol
Source: PLoS One. 2025 Oct 24;20(10):e0335151. doi: 10.1371/journal.pone.0335151 (PMC12551842; doi:10.1371/journal.pone.0335151)
Supplement: S2 Appendix — (DOCX) [file pone.0335151.s002.docx]

All code available at <https://github.com/mdcoblermd/MTP>

**Hyperparameter ranges tested by algorithm type:**

K-nearest neighbors

- N_neighbors: 2-810 by 2

Random forest

- N_estimators: 50, 100, 200
- Max_depth: None, 10, 20, 30
- Min_samples_split: 2,5,10
- Min_samples_leaf: 1,2,4

Logistic Regression

- C: 0.001, 0.01, 0.1, 1, 10, 100
- Penalty: L1, L2
- Solver: liblinear, saga
- Max_iter: 100, 200, 300

XGBoost

- Learning rate: 0.01, 0.05, 0.1
- Max_depth: 3, 5, 7
- Subsample: 0.6, 0.8, 1.0
- Colsample_bytree: 0.6, 0.8, 1.0
- N_estimators: 50, 100, 150, 300, 500

**Final XGBoost Model Hyperparameters chosen after optimization:**

MT-Arrival

- Learning rate: 0.05
- Max_depth: 5
- Subsample: 0.6
- Colsample_bytree: 0.6
- N_estimators: 500

MT-4 Hour

- Learning rate: 0.05
- Max_depth: 5
- Subsample: 0.6
- Colsample_bytree: 0.8
- N_estimators: 500

UMT-Arrival

- Learning rate: 0.05
- Max_depth: 3
- Subsample: 0.6
- Colsample_bytree: 1.0
- N_estimators: 500

UMT-4 Hour

- Learning rate: 0.05
- Max_depth: 5
- Subsample: 0.8
- Colsample_bytree: 0.8
- N_estimators: 500
