# Supplementary material for: MIASurviveMTP: Machine learning for immediate assessment and survival prediction after massive transfusion protocol
Source: PLoS One. 2025 Oct 24;20(10):e0335151. doi: 10.1371/journal.pone.0335151 (PMC12551842; doi:10.1371/journal.pone.0335151)
Supplement: S5 Appendix — (PDF) [file pone.0335151.s005.pdf]

MT Arrival AUROCs sorted by alternate missingness thresholds for removal of variable vs imputation:

66%

0.9004526405021113 95% Confidence Interval: [0.89301817 0.90760202]

50%

0.9012703127846147 95% Confidence Interval: [0.89400473 0.90989677]

33%

0.9026487068787142 95% Confidence Interval: [0.8954288 0.91085537]

20%

0.8918172509972777 95% Confidence Interval: [0.88412496 0.90079353]

10%

0.8455924324341332 95% Confidence Interval: [0.83677207 0.85609692]

MT 4-hour AUROCS sorted by alternate missingness thresholds for removal of variable vs imputation:

66%

0.9435122793459634 95% Confidence Interval: [0.93825923 0.94879428]

50%

0.9425955958012112 95% Confidence Interval: [0.93725901 0.94802108]

33%

0.9430911651241772 95% Confidence Interval: [0.93838923 0.94874331]

20%

0.938438354014888 95% Confidence Interval: [0.93343168 0.94440738]

10%

0.924056002001156 95% Confidence Interval: [0.91912895 0.93093728]

UMT Arrival AUROCs sorted by alternate missingness thresholds for removal of variable vs imputation:

66%

0.8588332126016365 95% Confidence Interval: [0.84693711 0.87378458]

50%

0.8577292682408078 95% Confidence Interval: [0.84628374 0.87218353]

33%

0.8595815301772941 95% Confidence Interval: [0.84827006 0.87292354]

20%

0.8457643192171082 95% Confidence Interval: [0.83374756 0.86152555]

10%

0.7858091463330718 95% Confidence Interval: [0.77076686 0.80186729]

UMT 4-hour AUROCS sorted by alternate missingness thresholds for removal of variable vs imputation:

66%

0.9195390488844517 95% Confidence Interval: [0.91177757 0.92945192]

50%

0.921754131463681 95% Confidence Interval: [0.91413789 0.93117147]

33%

0.9211363980417693 95% Confidence Interval: [0.91332185 0.93015324]

20%

0.9171666397678949 95% Confidence Interval: [0.9092463 0.9270388]

10%

0.8963546534249643 95% Confidence Interval: [0.88838814 0.90919702]
