# Supplementary material for: MIASurviveMTP: Machine learning for immediate assessment and survival prediction after massive transfusion protocol
Source: PLoS One. 2025 Oct 24;20(10):e0335151. doi: 10.1371/journal.pone.0335151 (PMC12551842; doi:10.1371/journal.pone.0335151)
Supplement: S6 Appendix — (PDF) [file pone.0335151.s006.pdf]

HTA Domain	Response from Study
	**Health Problem and Target Population**
**A clear description of the health problem and target population is required for intervention.**	Trauma-related hemorrhage requiring massive transfusion (MT) affects 3–5% of trauma patients and is a leading cause of early mortality. Patients undergoing MT (>5 units of blood in 4 hours) or UMT (>10 units of blood in 4 hours) are at high risk of death due to severe hemorrhage; these patients require rapid resuscitation and decision support.
**Definition of the health problem, including prevalence and incidence.**	MT is defined as >5 units of pRBC/WB in 4 hours; UMT is >10 units. An estimated 100,000 MT cases occur annually in the U.S.
**Information on the target population, such as mean age and risk factors.**	Target population includes trauma patients receiving MT; risk factors include hypotension, penetrating trauma, tachycardia, and positive Focused Assessment with Sonography in Trauma (FAST) exam. Trauma disproportionately affects young males, mean age of 40 in our study.
**Details on the standard therapeutic approach.**	Standard approach includes early activation of Massive Transfusion Protocols (MTP), using fixed ratios of blood products, but decisions on continuation or termination of resuscitation is currently guided only by clinical gestalt.
	**Technology Description**
**A detailed description of the technology and its context should be provided.**	The technology is a machine learning (ML) model predicting 6-hour mortality using TQIP data, built to support real-time decision of continuation or termination of massive resuscitation in patients for whom massive transfusion protocols have already been activated. It includes models using variables only available on arrival as well as models using variables available after 4 hours to simulated the addition of more clinical information to improved predictions as that information becomes available. All models are built using XGBoost, trained on TQIP data to simulate decision-making at different resuscitation phases.

HTA Domain	Response from Study
**Main characteristics of the technology and the needs it covers.**	Provides evidence-based mortality risk estimates during ongoing transfusion, helping clinicians stratify risk and potentially optimize blood product use, especially in low-resource situations such as low-acuity trauma center and mass casualty incidents.
**Intent to use, including intended and unintended use.**	Intended to guide but not replace clinician judgment in continuation of MTP; unintended use includes relying solely on model outputs to limit care.
**Technical evaluation and validation of the technology.**	Evaluated using AUROC, calibration curves, decision curve analysis, and SHAP interpretability; multiple models compared across timepoints. Internally validated on hold-out test set, but requires prospective external validation in future studies.
**Technical Aspects of Technology**	
**The technical evaluation focuses on the effectiveness and performance of the technology**	Uses an XGBoost predictive model to output a predicted probability of 6-hour mortality in a patient who is undergoing MTP, using variables available only on arrival (for arrival models) or variables available only after 4 hours (for 4-hour models). AUROC for MT models ranges from 0.901–0.943; for UMT 0.858–0.922. Outperforms traditional logistic regression, well-calibrated, and decision supportive. Will need prospective implementation to assess true clinical benefit.
**Training data used and their representativeness.**	Model trained on ACS TQIP 2017–2021 data (n≈30,000), covering hundreds of diverse trauma centers nationally.
**Model evaluation metrics and their clinical justification.**	Primary: AUROC (discrimination). Secondary: Brier score (calibration), F1 score, precision-recall AUC; chosen based on best practices for reporting predictive models in medicine.
**Robustness of the model and its transferability capacity.**	Robust to variable missingness thresholds (tested across 5 levels), missing value handling, and facility-specific differences. Also tested random imputation of key continuous variables for sensitivity analyses of impact of imputation with only modest performance degradation. Transferability

HTA Domain	Response from Study
	requires local prospective validation and will be the focus of future work.
**Traceability and records management to ensure transparency.**	All code is publicly available (S2 Appendix), full variable lists provided (S4 Appendix), and TRIPOD+AI checklist completed (S1 Appendix).
	**Technology Security**
**The risks associated with the use of technology are identified and assessed.**	Discusses risk of self-fulfilling prophecy and premature withdrawal of care; model outputs not intended to drive futility decisions. In regard to security risks, current models run without using PHI and have the capacity to be run locally.
**Clinical safety and risk assessment for patients and professionals.**	Model serves only to supplement decision-making. Use of conservative 6-hour mortality outcome reduces risk of overly pessimistic predictions.
**Technical security, including privacy and information quality.**	TQIP data is fully deidentified. No identifiable patient data involved at any stage.
	**Clinical Efficacy an Effectiveness**
**The clinical benefits of the technology under controlled and uncontrolled conditions are evaluated.**	Currently validated under retrospective conditions. AUROC for MT models ranges from 0.901–0.943; for UMT 0.858–0.922. Future prospective trials are needed to test efficacy in clinical workflows.
**Evidence of effectiveness to support expected benefits.**	Predictive models have shown strong performance across sensitivity analyses. No specific tools such as this have been previously developed for mortality prediction in MTP patients, but this tool does appear to outperform pre-existing tools like ABC score which has been applied to this use-case.
**Analysis of outcome variables according to the intended purpose.**	Primary outcome: AUROC. AUROC for MT models ranges from 0.901–0.943; for UMT 0.858–0.922
**Economic Aspects of Technology**	Economic impact not directly analyzed but has potential for improving blood stewardship, reducing waste, and ensuring

HTA Domain	Response from Study
	effective clinical interventions in resource-limited settings. This is a potential avenue for future work.
**The cost and cost-effectiveness of the technology are analyzed.**	Not yet analyzed; cost-benefit studies would be part of prospective implementation research.
**Comparison of acquisition, maintenance and use costs.**	Not included. Model is freely reproducible using open-source code. No proprietary software or costly infrastructure required.
**Evaluation of efficiency and use of resources compared to alternatives.**	No tools currently exist for this express purpose. Existing tools (e.g., ABC score) have been applied for this but perform poorly. Our model offers the first data-driven, evidence-based approach to a problem that is typically handled only by clinical gestalt.
