# Supplementary material for: MIASurviveMTP: Machine learning for immediate assessment and survival prediction after massive transfusion protocol
Source: PLoS One. 2025 Oct 24;20(10):e0335151. doi: 10.1371/journal.pone.0335151 (PMC12551842; doi:10.1371/journal.pone.0335151)
Supplement: Attachment — Submitted filename: responseLetter.docx [file pone.0335151.s007.docx]

Reviewer #1: This manuscript is a valuable attempt to address an important gap in trauma triage decision-making but is limited in its ability to provide reliable insights. I offer the following comments and suggestions for improvement:

1. A striking finding in this work is that missing data itself emerged as the top predictor of mortality in several models. Specifically, SHAP analysis showed that the absence of a recorded height was the single most impactful feature for MT on arrival and both UMT models. The lack of an ED drug screen was highly impactful in the MT 4-hour model. This probably indicates that critically ill patients who are near death do not get certain routine data collected (height, weight, drug testing), making “missingness” a proxy for severity of injury or urgency of care. This is a reasonable predictive signal and is consistent with previous observational studies (for example, Donohue et al. 2024 note that missing laboratory values carried prognostic information PMID: 38375027). However, it raises critical issues for interpretation and implementation. The authors should note that the model does not imply that a patient’s body height or weight causes mortality, but rather that not having those charted is a red flag (likely because the team was too busy resuscitating to document them). Stating this distinction is important to avoid misinterpretation of the feature importance. At minimum, a robust discussion of this issue is warranted as these findings do not provide physiological insight and could be viewed as a form of “algorithmic workaround.”. However, ideally, the authors should assess the stability of such predictors: if in the future hospitals enforce entering a height/weight by default (or, for instance, auto-fill from driver’s license, etc.), that predictive signal might disappear. How would the model perform then? As a suggestion, the authors might explore an experiment: remove or impute the “missing height/weight” in different ways and see how much the model AUROC drops. If performance drops just a little, it means the model’s accuracy is not entirely dependent on this artifact; if it drops a lot, that implies the model is leveraging process-of-care signals heavily. Apart from this, it would also be useful to mention any steps taken to handle missing data during training in general. For example, did you use imputation for variables or rely on tree-based algorithms’ ability to handle missing values natively? In short, the case of addressing missingness here is such a critical issue that its handling would affect the quality of all downstream analyses. Therefore, all remaining comments are of secondary importance.

**Thank you for these comments. We agree that models relying heavily on missingness for their predictions can be inherently fragile, especially when moving to implementation between specific sites. In light of this, all binary missingness indicators have been removed from all models. Feature analysis demonstrates that imputed values for height and weight are still being picked up on by the model as a proxy for missingness and therefore acuity, though these variables are significantly less impactful than they were when missingness was explicitly modeled. To demonstrate the stability of the models without any indicators for missingness, separate models were subsequently ran where height, weight, and temperature were all imputed randomly using normally distributions matching the parameters of each of these respective variables within the training set. While this did lead to performance decrease, the models still perform well, and the performance degradation is relatively minor at the 4-hour timepoint. All-in-all, this points towards the need to assess model stability and performance at an individual site before, during, and after implementation and may necessitate model choices that are customized to that site depending on the facilities culture, practices, and capabilities. Substantial text has been added to the discussion to address these points regarding custom implementation, especially focusing on the utility of missingness (lines 336-355). We also added further content to the portion of the discussion focused on warning about the utility of feature interpretation from a SHAP analysis (lines 322-335). Finally, language was added to the methods section to describe data imputation methods (lines 127-234).**

2. The authors mention conventional MTP activation scores and note their modest performance for mortality prediction (60–70% AUROC). It would strengthen the background to emphasize how this study’s focus on mortality after MTP initiation differs from the more common focus on predicting the need for MTP. Recent work by Benjamin et al. developed an ML model to predict massive transfusion requirements in trauma (PMID: 37746616). By contrast, the present study targets patients already receiving MT, aiming to predict who will not survive despite transfusion, which is a novel angle. Highlighting this distinction and the clinical rationale (e.g., informing resource allocation) would help readers identify the study’s unique contribution. Additionally, the 6-hour mortality as the result is not the most common choice (reporting 24-hour or in-hospital mortality is more frequent). The manuscript would benefit from clearer justification. Presumably, this timeframe captures patients who exsanguinate or die from uncontrollable hemorrhage shortly after admission, and the intent is to aid in real-time futility decisions. If so, the authors may want to state this explicitly. If there are relevant references or guidelines on early prognostication in massive hemorrhage or “futility” thresholds, they should be cited. Additionally, since the authors have data on 30-day outcomes, it might be informative to discuss how 6-hour mortality relates to longer-term mortality in this cohort. (Many patients who die within 6 hours likely account for a large fraction of 24-hour and 30-day deaths, as your data show 22% 6h mortality vs 41% 30-days). Clarifying this helps readers understand the scope and limits of the model (it probably does not predict longer-term complications or late deaths, only the outcome of the immediate resuscitation phase). The authors should consider comparing the performance of their models for 6-hour mortality to their performance (or a separate model) for 24-hour or in-hospital mortality. This could show whether the feature importance or accuracy changes when predicting a later endpoint.

**Additional commentary was added to the discussion to reflect the fact that prior models have focused on predicting who will require massive transfusion activation, not prediction of mortality after activation has occurred, which is the novelty of our model, in addition to potential benefit this could lead to (lines 285-287). Additionally, justification of the 6-hour mortality outcomes was added (lines 137-43). We use the 6-hour timepoint as this is frequently reported in many hemorrhage-control-intervention related studies, and this reflects the most conservative estimate of mortality (as mortality will get progressively higher at more distant timepoints). Additionally, later mortality timepoints would be more affected by specific treatments administered to the patient after the initial resuscitation as opposed to the patient’s presenting condition itself. More information regarding prior work on futility in massive transfusion was also added (lines 277-284).**

3. The inclusion criteria and definitions for MT and UMT should be clearly reiterated, and any potential biases should be acknowledged. You define MT as >5 units PRBC (+-WB) in 4 hours, which is a reasonable operational definition, and UMT as >10 units in 4 hours. One concern is that by requiring patients to receive these large transfusion volumes, the study inherently excludes patients who died extremely rapidly before reaching the MT threshold. For example, a patient who hemorrhaged and died after 4 units in 1 hour would not meet “MT” by your definition and thus would be omitted from the analysis. This could bias the cohort toward survivors (i.e., those who lived long enough to get 5+ units), potentially inflating predictive performance. Studies on mortality cutoffs by transfusion volume may be worth citing. For example, Clements et al. 2023 found no absolute “futility” transfusion cutoff, meaning that even patients requiring extremely high units sometimes survive (PMID 37125814). The Methods note the exclusion of cases with missing mortality data, but the authors could also discuss this survivor bias issue in the Limitations section if applicable. The present model, by focusing on those who did receive MT, is aimed at identifying who among them will die, but not those who die before MT can be completed. On a related note, if there were any substantial differences in patient characteristics or outcomes between earlier years and later years (when whole blood became available), the authors should consider mentioning whether the model performance was consistent across time.

**Thank you for pointing out this limitation. We have added a section discussing survivorship bias to the discussion and mentioned that this model would therefore not be applicable to patients who do not receive at least 5 units of blood, even if the patient had a massive transfusion protocol activation (lines 318-321). The use of whole blood separately was removed as a variable in the model as it was only available for patients in the later 2 years of the database utilized. Instead, we sum the total red-blood cell containing products and utilize that value instead of separating out by whole blood and packed red blood cells.**

4. The authors acknowledge that external validation is lacking, and this is a critical limitation for any prediction model intended for broad use. They can consider actionable steps to address it. For instance, if any external trauma data set is available (such as a single-center data from a different hospital system), testing the model there would substantially strengthen the paper. If this revision is not feasible, the authors should at least discuss plans for prospective validation.

**A section was added about future plans for prospective validation (lines 379-387).**

5. In decision curve analysis, the authors should explain what threshold range might be reasonable for a trauma team to act upon. For instance, if the model predicts >50% risk of 6h mortality, is that a point where one might consider stopping aggressive intervention? Or would a lower threshold (say 20–30% risk) trigger some changes in management?

**A section was added to discuss how site-specific practices will need to be evaluated along with model performance at individual sites to determine optimal decision thresholds and interventions that should accompany those decision thresholds, as both of these are heavily dependent on site-specific capabilities, practice patterns, and standards of care (lines 369-376).**

6. The authors are encouraged to compare their ML model’s performance to existing predictive models or scores in trauma to put the results in context. In the Introduction, they cite that the ABC score and Massive Transfusion Score achieved only around 0.65–0.70 AUROC for mortality after MT. Were those numbers derived from prior literature, or did you compute them from your dataset? On the same note, TRISS is a logistic model combining physiology, injury anatomy, and age to predict survival. While TRISS is not specific to massive transfusion patients, it’s a widely known comparator. It would be informative to see how a traditional approach like TRISS or an ISS-based logistic regression performs on the MT cohort for predicting 6h or 30d mortality. Perhaps the authors could compute an ISS + vital signs logistic model as a baseline. This would show readers what improvement the ML model offers beyond what could be done with a simple regression on known predictors.

**Thank you for this suggestion. One of the benefits of this type of model is that the results are derivable in real-time. ISS, and therefore any metric using ISS (such as TRISS), is not derivable in real time, and therefore comparing our models’ predictions to ISS would not provide any useful information for real-time decision making. We do however compare our model to a L1 regularized regression model that has access to the same variables as the decision-tree model and note that the decision-tree model does outperform the regression. We have added wording to note the limitation of not being able to calculate ABC or Massive Transfusion Scores in our specific dataset due to lack of variables needed for those scores (lines 313-315).**

7. I suggest the manuscript add more detail on the final model specifications, such as reporting the chosen hyperparameters for the GBDT model and optimization choice.

**This information was added explicitly to the S2 supplement.**

8. Given the choice of study population and outcomes, it would be prudent to warn against misinterpretation in the discussion: An output of “90% mortality risk” could become a self-fulfilling prophecy if it unduly biases providers to withdraw care. Perhaps the model is most useful for system-level decisions (like alerting the blood bank) rather than bedside decisions to stop care. The authors might mention how the action threshold may vary by institution.

**We agree this was understated in the original version of the manuscript. An entire paragraph has been added to the discussion to explicitly warn about self-fulfilling prophecy and potential consequences and more completely discuss the potential role for this model (lines 365-376).**

9. The authors should consider discussing how the model could be enhanced or extended. One clear path is incorporating more data modalities. Prior studies in surgical prediction have shown that adding unstructured text can improve performance. For a trauma patient, surgeon’s assessment or EMS reports, as well as radiographic results (FAST exam, CT scans) could further refine mortality prediction. Another direction is to develop a dynamic or sequential model. Instead of just two static time points (arrival and 4h), one could use a time-series approach (e.g., updating the risk after each hour or after each significant event in the resuscitation). Some patients (especially in trauma) might die of TBI or other non-hemorrhagic causes even if hemorrhage is controlled. Could the model be refined to differentiate hemorrhage-related mortality? For example, integrating measures of head injury (CT findings) may allow a sense of whether the predicted death is due to unrevivable brain trauma versus ongoing hemorrhage. This could guide whether to continue transfusion or not.

**Our goal with this model is to lay the groundwork for future models that are able to harness even better predictors like these mentioned above and therefore able to improve predictions further. We agree that future models should focus on expanding to include these more granular predictors We have added a section discussing plans for model extension that will be occurring simultaneously with our planned prospective validation (lines 387-393).**

These final ideas may be beyond the scope of implementation now and should only be addressed after the fundamental methodologic shortcomings have been rectified.
