# Supplementary material for: MIASurviveMTP: Machine learning for immediate assessment and survival prediction after massive transfusion protocol
Source: PLoS One. 2025 Oct 24;20(10):e0335151. doi: 10.1371/journal.pone.0335151 (PMC12551842; doi:10.1371/journal.pone.0335151)
Supplement: Attachment — Submitted filename: responseLetter_r2.docx [file pone.0335151.s009.docx]

Additional Editor Comments:

Page 4 line 87 replace “achieved” with “achieve”.

**We thank the editor for catching this typo and have corrected it.**

Reviewer’s Responses to Questions

Comments to the Author

1. If the authors have adequately addressed your comments in a previous round of review and you feel that this manuscript is now acceptable for publication, you may indicate that here to bypass the “Comments to the Author” section, enter your conflict of interest statement in the “Confidential to Editor” section, and submit your “Accept” recommendation.

Reviewer #2: All comments have been addressed

**Thank you to all involved reviewers and editors for the time spent reviewing our manuscript and all constructive criticism. We believe it is a much stronger paper as a result.**
